# Supplementary material for: A two-stage random-effects estimator for meta-analyses of the value per statistical life
Source: PLoS One. 2025 Jun 13;20(6):e0324630. doi: 10.1371/journal.pone.0324630 (PMC12165433; doi:10.1371/journal.pone.0324630)
Supplement: S0 Graphical abstract — (PDF) [file pone.0324630.s001.pdf]

Graphical abstract

We developed a **two-stage meta-analysis estimator** for synthesizing effect sizes published in independent studies. The primary estimates from each study are used to compute study-level means, then the study-level means are used to compute an overall mean. Sampling error variances (based on standard errors reported in each study) and non-sampling error variances (estimated from the meta-data) are used to compute efficient weights for combining the estimates.

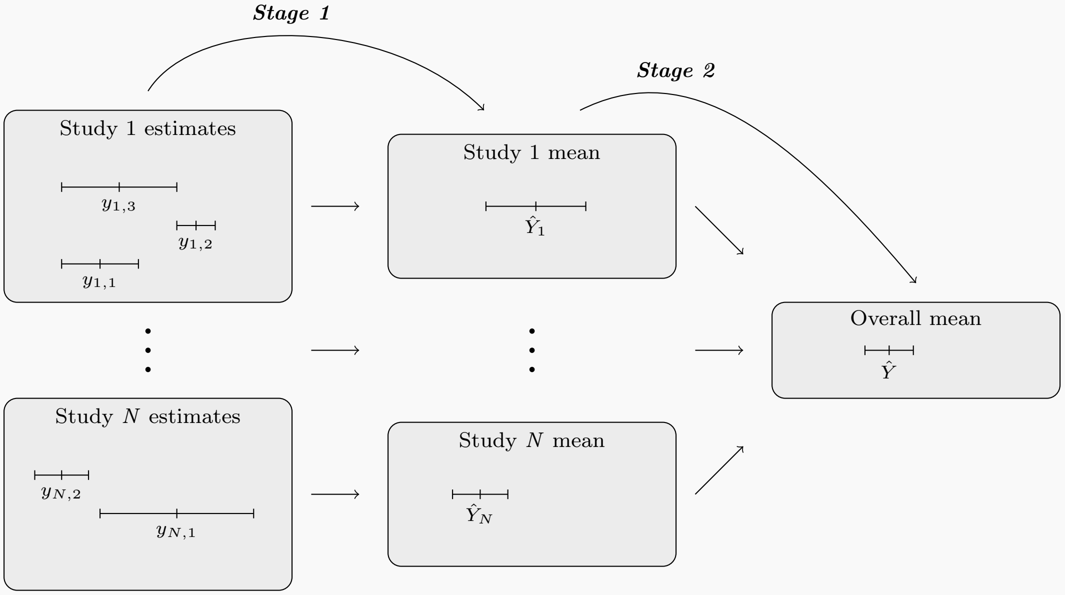

We used simulation tests to compare the performance of our estimator to other commonly used meta-analysis methods, and we demonstrated our approach in an application to a real meta-dataset including 113 estimates of the **value per statistical life** (VSL) drawn from 19 published studies.
