# Supplementary material for: A two-stage random-effects estimator for meta-analyses of the value per statistical life
Source: PLoS One. 2025 Jun 13;20(6):e0324630. doi: 10.1371/journal.pone.0324630 (PMC12165433; doi:10.1371/journal.pone.0324630)
Supplement: S1 Appendix — Derivation of two-stage random-effects estimator. (PDF) [file pone.0324630.s002.pdf]

Supporting Information

S1 Appendix. In this appendix we derive the two-stage random-effects estimator used in the main text. The dataset is composed of $i = 1, 2, ..., I$ groups of observations, where group i is composed of $j = 1, 2, ..., J_i$ individual observations. (The primary grouping strategy in our application groups estimates based on the same underlying dataset, but other strategies are possible, e.g., grouping by study or primary author. The aim is to group observations such that correlations across groups are minimized.) We will denote the observations as y_{ij} and their associated standard errors as se_{ij}. The estimator will take the form of a weighted mean, $\hat{Y} = \sum_{i=1}^{I} \sum_{j=1}^{J_i} w_{ij} y_{ij}$, where the weights sum to one, $\sum_{i=1}^{I} \sum_{j=1}^{J_i} w_{ij} = 1$. Our task is to find the optimal weights given the structure of our data set and our assumptions about the nature of the data generating process.

S1.1 Sources of error

We decompose each observation into the sum of the true mean and three error components:

$$y_{ij} = Y + \eta_i + \mu_{ij} + \varepsilon_{ij}, \tag{1}$$

where Y is the true value of the average VSL among the U.S. adult general population (our target of estimation), η_i is a group-level non-sampling error, μ_{ij} is an observation-level non-sampling error, and ε_{ij} is an observation-level sampling error. η_i varies among but not within groups, while both μ_{ij} and ε_{ij} vary both among and within groups. (Below we describe how to generalize this meta-analysis model to a linear meta-regression model, which basically involves replacing y_{ij} with $x_{ij}\beta$ throughout.)

Before proceeding with the derivation, it might be helpful to explain our assumptions about the nature of the error components. Sampling errors, represented by ε_{ij}, arise from sampling variability alone. This refers to the variability of a statistic if it were calculated many times repeating the same study design with the same sample size but with a different random draw of observations from the target population each time. Non-sampling errors include all other sources of deviation between the estimate calculated from the sample and the true quantity that is the target of estimation, such as measurement error, missing variable bias, other forms of model mis-specification, mis-matches between the sampling frame and the population of interest, ad hoc treatment of "outliers," and other methodological choices that may lead to biased estimates. The practical relevance of this distinction is that the standard errors reported in the original studies represent only the sampling variability of the primary VSL estimates. Therefore, these quantities can serve as estimates of the standard deviations of ε_{ij} but not the other error components in the model, the variances of which must be estimated using the meta-data itself.

We will assume that the non-sampling error components are uncorrelated with each other and with the sampling errors, but we will allow for possible correlations among sampling errors within groups. Also note that the composite non-sampling errors, $\eta_i + \mu_{ij}$, will be correlated within groups but not across groups by the assumption that η_i is common to all observations in group i. We will derive the minimum variance estimator and calculate the associated standard error based on these assumptions. We also will calculate standard errors using both a bootstrap approach and the robust standard errors proposed by Hedges et al. [1] to avoid the bias of nominal standard errors when the assumed error structure does not correspond to the true error structure.

S1.2 A two-stage random effects estimator

To find the optimal weights to place on each observation in the meta-dataset we proceed in two stages. In the first stage we find the optimal feasible weights, $\hat{g}_{ij}$, for calculating composite estimates for each group,

$$\hat{Y}_i = \sum\nolimits_{j=1}^{J_i} \hat{g}_{ij} y_{ij}. \tag{2}$$

We use " ^ " notation to indicate quantities that can be computed from our data or other estimated quantities. The same symbol without a " ^ " indicates an unknown population parameter or an infeasible estimator because it depends on one or more unknown population parameters.

In the first stage we impose the constraint that $\sum_{j=1}^{J_i} \hat{g}_{ij} = 1$, which is required to make the group-level composite estimate an unbiased estimator of the group mean, $Y + \eta_i$. In the second stage we find the optimal weights, $\hat{h}_i$, for calculating the overall composite estimate,

$$\hat{Y} = \sum\nolimits_{i=1}^{I} \hat{h}_i \hat{y}_i. \tag{3}$$

In this stage we impose the constraint that $\sum_{i=1}^{I} \hat{h}_i = 1$, which is required to make the expected value of the composite estimate equal to the mean of the group-level effects, and therefore equal to the true effect Y by the assumption that the expected value of the group level non-sampling errors η_i is zero. The composite weights for each observation are then $\hat{w}_{ij} = \hat{h}_i \hat{g}_{ij}$.

S1.3 Stage one

We begin by finding the optimal infeasible weights for calculating the group-level composite estimates, $\hat{Y}_i$. We will denote the variance of the composite estimate for group i as v_i, which is

$$v_i = \mathbb{E}\left[\left(\sum\nolimits_{j=1}^{J_i} g_{ij} y_{ij}\right)^2\right] - \mathbb{E}\left[\left(\sum\nolimits_{j=1}^{J_i} g_{ij} y_{ij}\right)\right]^2. \tag{4}$$

Substituting $y_{ij} = Y + \eta_i + \mu_{ij} + \varepsilon_{ij}$ gives

$$v_i = \mathbb{E}\left[\left(\sum\nolimits_{j=1}^{J_i} g_{ij} \left(Y + \eta_i + \mu_{ij} + \varepsilon_{ij}\right)\right)^2\right] - \mathbb{E}\left[\left(\sum\nolimits_{j=1}^{J_i} g_{ij} \left(Y + \eta_i + \mu_{ij} + \varepsilon_{ij}\right)\right)\right]^2. \tag{5}$$

Using the constraint $\sum_{j=1}^{J_i} g_{ij} = 1$, we can factor the Y and η_i out of the summation in the first term, and we can simplify the second term to Y^2, which gives

$$v_i = \mathbb{E}\left[\left(Y + \eta_i + \sum\nolimits_{j=1}^{J_i} g_{ij} \left(\mu_{ij} + \varepsilon_{ij}\right)\right)^2\right] - Y^2. \tag{6}$$

By assumption, η_i is uncorrelated with both μ_{ij} and ε_{ij}, so all terms involving products of η_i and μ_{ij} or η_i and ε_{ij} will equal zero in expectation. All terms involving products of Y and any error terms also will equal zero in expectation, so we can simplify equation (6) to

$$v_i = \mathbb{E}\left[(Y + \eta_i)^2 + \left(\sum\nolimits_{j=1}^{J_i} g_{ij} \left(\mu_{ij} + \varepsilon_{ij}\right)\right)^2\right] - Y^2. \tag{7}$$

Completing both squares inside the expectation operator and eliminating the Y^2 and $-Y^2$ terms gives

$$v_i = \mathbb{E}\left[\eta_i^2 + 2y\eta_i + \sum_{j=1}^{J_i} \left\{ g_{ij}^2 \left(\mu_{ij} + \varepsilon_{ij}\right)^2 + \sum_{k \neq j}^{J_i} g_{ij} g_{ik} \left(\mu_{ij} + \varepsilon_{ij}\right)\left(\mu_{ik} + \varepsilon_{ik}\right) \right\} \right], \quad (8)$$

where $\sum_{k\neq j}^{J_i}$ indicates the sum from 1 to J_i excluding element j. Note that $\mathbb{E}\big[\big(\mu_{ij}^2 + \varepsilon_{ij}^2\big)\big]$ is the variance of observation ij, and $\mathbb{E}\big[\big(\mu_{ij} + \varepsilon_{ij}\big)\big(\mu_{ik} + \varepsilon_{ik}\big)\big]$ is the covariance between observation ij and ik (both conditional on η_i).

Next we evaluate the right hand side of equation (8) using the assumptions that all error terms are mean zero and all but the sampling errors are uncorrelated, and using the fact that the covariance between two random variables equals their correlation multiplied by their respective standard deviations. This gives

$$v_i = \sigma_\eta^2 + \sum_{j=1}^{J_i} \left[g_{ij}^2 \left(\sigma_{\mu,i}^2 + se_{ij}^2\right) + \rho_i \sum_{k\neq j}^{J_i} g_{ij} g_{ik} se_{ij} se_{ik} \right], \quad (9)$$

where ρ_i is the correlation among sampling errors for observations in group i. Equation (9) is the quantity we want to minimize by choosing weights, g_{ij}, subject to the constraint that the weights sum to one. The Lagrangian is

$$\mathcal{L}_i = \sigma_\eta^2 + \sum_{j=1}^{J_i} \left[g_{ij}^2 \left(\sigma_{\mu,i}^2 + se_{ij}^2\right) + \rho_i \sum_{k\neq j}^{J_i} g_{ij} g_{ik} se_{ij} se_{ik} \right] - \lambda_i \left(\sum_{j=1}^{J_i} g_{ij} - 1 \right), \quad (10)$$

and the first-order conditions for a minimum are

$$\frac{\partial \mathcal{L}_i}{\partial g_{ij}} = 2g_{ij}\left(\sigma_{\mu,i}^2 + se_{ij}^2\right) + 2\rho_i \sum_{k\neq j}^{J_i} g_{ik} se_{ij} se_{ik} - \lambda_i = 0, \quad (11)$$

for each j in group i.

If it is not obvious where the 2 multiplying ρ_i in equation (11) comes from, note that in equation (10) the double summation term, $\sum_{j=1}^{J_i}\sum_{k\neq j}^{J_i} g_{ij} g_{ik} se_{ij} se_{ik}$, is the sum of all off-diagonal elements of the $J_i \times J_i$ matrix formed by cross-multiplying the vector $\mathbf{g}_i \odot \mathbf{se}_i$ by itself, where $\odot$ indicates element-by-element multiplication, i.e., $[\mathbf{g}_i \odot \mathbf{se}_i]'[\mathbf{g}_i \odot \mathbf{se}_i] = \sum_{j=1}^{J_i}\sum_{k=1}^{J_i} g_{ij} g_{ik} se_{ij} se_{ik}$. Taking the derivative of the sum of the off-diagonal terms with respect to any given element of the vector $\mathbf{g}_i$ gives 2 times the sum of all other elements of $\mathbf{g}_i$ because each of these elements appears once below and once above the diagonal of $[\mathbf{g}_i \odot \mathbf{se}_i]'[\mathbf{g}_i \odot \mathbf{se}_i]$.

Next, subtracting $2\rho_i \sum_{k\neq j}^{J_i} g_{ik} se_{ij} se_{ik} - \lambda_i$ from both sides of the second equality in expression (11) gives

$$2g_{ij}\left(\sigma_{\mu,i}^2 + se_{ij}^2\right) = \lambda_i - 2\rho_i \left(-g_{ij} se_{ij}^2 + \sum_{k=1}^{J_i} g_{ik} se_{ij} se_{ik} \right), \quad (12)$$

and then distributing the $2\rho_i$ to the terms inside the parentheses on the right hand side of equation (12) gives

$$2g_{ij}\left(\sigma_{ij}^2 + se_{ij}^2\right) = \lambda_i + 2\rho_i g_{ij} se_{ij}^2 - 2\rho_i \sum_{k=1}^{J_i} g_{ik} se_{ij} se_{ik}. \quad (13)$$

Next, we subtract $2\rho_i g_{ij} se_{ij}^2$ from both sides of equation (13) to get

$$2g_{ij}\left(\sigma_{\mu,i}^2 + se_{ij}^2\right) - 2\rho_i g_{ij} se_{ij}^2 = \lambda_i - 2\rho_i \sum_{k=1}^{J_k} g_{ik} se_{ij} se_{ik}. \quad (14)$$

Then we factor $2g_{ij}$ out of the left hand side of equation (14) to get

$$2g_{ij}\left[\left(\sigma_{\mu,i}^2 + se_{ij}^2\right) - \rho_i se_{ij}^2\right] = \lambda_i - 2\rho_i \sum_{k=1}^{J_i} g_{ik} se_{ij} se_{ik}, \quad (15)$$

then solve for g_{ij} by dividing both sides of equation (15) by $2\left[\left(\sigma_{\mu,i}^2 + se_{ij}^2\right) - \rho_i se_{ij}^2\right]$, which gives

$$g_{ij} = \frac{\lambda_i - 2\rho_i se_{ij} \sum_{k=1}^{J_i} se_{ik} g_{ik}}{2\left[\sigma_{\mu,i}^2 + (1-\rho_i)\, se_{ij}^2\right]}. \tag{16}$$

Next, we apply the constraint that the g_{ij}'s must sum to 1 in each group to get

$$1 = \sum_{j=1}^{J_i} \frac{\lambda_i - 2\rho_i se_{ij} \sum_{k=1}^{J_i} se_{ik} g_{ik}}{2\left[\sigma_{\mu,i}^2 + (1-\rho_i)\, se_{ij}^2\right]}, \tag{17}$$

and then separate the term on the right hand side of equation (17) into two sums:

$$1 = \sum_{j=1}^{J_i} \frac{\lambda_i}{2\left[\sigma_{\mu,i}^2 + (1-\rho_i)\, se_{ij}^2\right]} - \sum_{j=1}^{J_i} \frac{2\rho_i se_{ij} \sum_{k=1}^{J_i} se_{ik} g_{ik}}{2\left[\sigma_{\mu,i}^2 + (1-\rho_i)\, se_{ij}^2\right]}. \tag{18}$$

Next we factor the Lagrange multiplier out of the sum in the first term and cancel the 2's in the second term on the right hand side of equation (18) to get

$$1 = \lambda_i \sum_{j=1}^{J_i} \frac{1}{2\left[\sigma_{\mu,i}^2 + (1-\rho_i)\, se_{ij}^2\right]} - \sum_{j=1}^{J_i} \frac{\rho_i se_{ij} \sum_{k=1}^{J_i} se_{ik} g_{ik}}{\left[\sigma_{\mu,i}^2 + (1-\rho_i)\, se_{ij}^2\right]}. \tag{19}$$

We solve for the Lagrange multiplier by adding the second term on the right hand side to both sides of equation (19) then dividing both sides by the term that multiplies the Lagrange multiplier:

$$\lambda_i = \frac{1 + \sum_{j=1}^{J_i} \frac{\rho_i se_{ij} \sum_{k=1}^{J_i} se_{ik} g_{ik}}{\left[\sigma_{\mu,i}^2 + (1-\rho_i) se_{ij}^2\right]}}{\sum_{j=1}^{J_i} \frac{1}{2\left[\sigma_{\mu,i}^2 + (1-\rho_i) se_{ij}^2\right]}}. \tag{20}$$

Next, we rearrange equation (20) by factoring $\rho_i se_{ij} \sum_{k=1}^{J_i} se_{ik} g_{ik}$ out of the sum over j in the numerator and substituting $\frac{1}{\left[\sigma_{\mu,i}^2 + (1-\rho_i) se_{ij}^2\right]} = \left[\sigma_{\mu,i}^2 + (1-\rho_i)\, se_{ij}^2\right]^{-1}$ in both the numerator and denominator of (20) to get

$$\lambda_i = \frac{1 + \left(\rho_i se_{ij} \sum_{k=1}^{J_i} se_{ik} g_{ik}\right) \sum_{j=1}^{J_i} \left[\sigma_{\mu,i}^2 + (1-\rho_i)\, se_{ij}^2\right]^{-1}}{\frac{1}{2} \sum_{j=1}^{J_i} \left[\sigma_{\mu,i}^2 + (1-\rho_i)\, se_{ij}^2\right]^{-1}}. \tag{21}$$

Then we plug (21) back into the expression for g_{ij} in equation (16) and divide the numerator and denominator by 2 to get

$$g_{ij} = \frac{\frac{1 + \left(\rho_i se_{ij} \sum_{k=1}^{J_i} se_{ik} g_{ik}\right) \sum_{j=1}^{J_i} \left[\sigma_{\mu,i}^2 + (1-\rho_i) se_{ij}^2\right]^{-1}}{\sum_{j=1}^{J_i} \left[\sigma_{\mu,i}^2 + (1-\rho_i) se_{ij}^2\right]^{-1}} - \rho_i se_{ij} \sum_{k=1}^{J_i} se_{ik} g_{ik}}{\sigma_{\mu,i}^2 + (1-\rho_i)\, se_{ij}^2} \tag{22}$$

which we can separate into two fractions,

$$g_{ij} = \frac{1 + \left(\rho_i se_{ij} \sum_{k=1}^{J_i} se_{ik} g_{ik}\right) \sum_{j=1}^{J_i} \left[\sigma_{\mu,i}^2 + (1-\rho_i)\, se_{ij}^2\right]^{-1}}{\left[\sigma_{\mu,i}^2 + (1-\rho_i)\, se_{ij}^2\right] \sum_{j=1}^{J_i} \left[\sigma_{\mu,i}^2 + (1-\rho_i)\, se_{ij}^2\right]^{-1}} - \frac{\rho_i se_{ij} \sum_{k=1}^{J_i} se_{ik} g_{ik}}{\left[\sigma_{\mu,i}^2 + (1-\rho_i)\, se_{ij}^2\right]}, \tag{23}$$

then we separate the first term on the right hand side into two fractions and rearrange terms in the sum to get:

$$g_{ij} = \frac{1}{\sum_{k=1}^{J_i} \frac{\sigma_{\mu,i}^2 + (1-\rho_i) se_{ij}^2}{\sigma_{\mu,i}^2 + (1-\rho_i) se_{ik}^2}} + \frac{\left(\rho_i \sum_{k=1}^{J_i} se_{ik} g_{ik}\right) \sum_{k=1}^{J_i} \frac{se_{ik}}{\sigma_{\mu,i}^2 + (1-\rho_i) se_{ik}^2}}{\sum_{k=1}^{J_i} \frac{\sigma_{\mu,i}^2 + (1-\rho_i) se_{ij}^2}{\sigma_{\mu,i}^2 + (1-\rho_i) se_{ik}^2}} -$$

$$\frac{\rho_i se_{ij} \sum_{k=1}^{J_i} se_{ik} g_{ik}}{\left[\sigma_{\mu,i}^2 + (1-\rho_i)\, se_{ij}^2\right]}. \tag{24}$$

Then we move $\rho_i \sum_{k=1}^{J_i} se_{ik} g_{ik}$ out of the numerator of both fractions in which it appears to get

$$g_{ij} = \frac{1}{\sum_{k=1}^{J_i} \frac{\sigma_{\mu,i}^2 + (1-\rho_i) se_{ij}^2}{\sigma_{\mu,i}^2 + (1-\rho_i) se_{ik}^2}} + \left(\frac{\sum_{k=1}^{J_i} \frac{se_{ik}}{\sigma_{\mu,i}^2 + (1-\rho_i) se_{ik}^2}}{\sum_{k=1}^{J_i} \frac{\sigma_{\mu,i}^2 + (1-\rho_i) se_{ij}^2}{\sigma_{\mu,i}^2 + (1-\rho_i) se_{ik}^2}} \right) \rho_i \sum_{k=1}^{J_i} se_{ik} g_{ik} - \frac{se_{ij}}{\sigma_{\mu,i}^2 + (1-\rho_i)\, se_{ij}^2} \rho_i \sum_{k=1}^{J_i} se_{ik} g_{ik} \tag{25}$$

Then we distribute terms into the summations in the second and third terms on the right hand side of equation (25) to get

$$g_{ij} = \frac{1}{\sum_{k=1}^{J_i} \frac{\sigma_{\mu,i}^2 + (1-\rho_i) se_{ij}^2}{\sigma_{\mu,i}^2 + (1-\rho_i) se_{ik}^2}} + \rho_i \sum_{k=1}^{J_i} \left(\frac{\sum_{k=1}^{J_i} \frac{se_{ik}}{\sigma_{\mu,i}^2 + (1-\rho_i) se_{ik}^2}}{\sum_{k=1}^{J_i} \frac{\sigma_{\mu,i}^2 + (1-\rho_i) se_{ij}^2}{\sigma_{\mu,i}^2 + (1-\rho_i) se_{ik}^2}} \right) se_{ik} g_{ik} - \rho_i \sum_{k=1}^{J_i} \left(\frac{se_{ij}}{\sigma_{\mu,i}^2 + (1-\rho_i)\, se_{ij}^2} \right) se_{ik} g_{ik}. \tag{26}$$

Next, we combine the second and third terms into a single summation to get

$$g_{ij} = \frac{1}{\sum_{k=1}^{J_i} \frac{\sigma_{\mu,i}^2 + (1-\rho_i) se_{ij}^2}{\sigma_{\mu,i}^2 + (1-\rho_i) se_{ik}^2}} + \rho_i \sum_{k=1}^{J_i} \left(\frac{\sum_{k=1}^{J_i} \frac{se_{ik}}{\sigma_{\mu,i}^2 + (1-\rho_i) se_{ik}^2}}{\sum_{k=1}^{J_i} \frac{\sigma_{\mu,i}^2 + (1-\rho_i) se_{ij}^2}{\sigma_{\mu,i}^2 + (1-\rho_i) se_{ik}^2}} - \frac{se_{ij}}{\sigma_{\mu,i}^2 + (1-\rho_i)\, se_{ij}^2} \right) se_{ik} g_{ik}. \tag{27}$$

Equation (27) has the form of

$$g_{ij} = A_{ij} + \sum_{k=1}^{J_i} B_{ijk} g_{ik}, \tag{28}$$

where

$$A_{ij} = \frac{1}{\sum_{k=1}^{J_i} \frac{\sigma_{\mu,i}^2 + (1-\rho_i) se_{ij}^2}{\sigma_{\mu,i}^2 + (1-\rho_i) se_{ik}^2}} \tag{29}$$

and

$$B_{ijk} = \rho_i \left(\frac{\sum_{\ell=1}^{J_i} \frac{se_{i\ell}}{\sigma_{\mu,i}^2 + (1-\rho_i) se_{i\ell}^2}}{\sum_{\ell=1}^{J_i} \frac{\sigma_{\mu,i}^2 + (1-\rho_i) se_{ij}^2}{\sigma_{\mu,i}^2 + (1-\rho_i) se_{i\ell}^2}} - \frac{se_{ij}}{\sigma_{\mu,i}^2 + (1-\rho_i)\, se_{ij}^2} \right) se_{ik}. \tag{30}$$

Equation (28) can be written in matrix notation as

$$\mathbf{g}_i = \mathbf{A}_i + \mathbf{B}_i \mathbf{g}_i, \tag{31}$$

so, finally, we can solve for $\mathbf{g}_i$ as follows:

$$\left(\mathbf{I}_i - \mathbf{B}_i\right) \mathbf{g}_i = \mathbf{A}_i \Rightarrow \mathbf{g}_i = \left(\mathbf{I}_i - \mathbf{B}_i\right)^{-1} \mathbf{A}_i. \tag{32}$$

S1.4 Cross checks

To provide some indirect confirmation that the above derivation is valid, we can apply the formula for the optimal weights to two simple cases where the weights are straightforward to verify independently. First, consider the case when the sampling errors in a group are uncorrelated. When $\rho_i = 0$, equation (30) implies $\mathbf{B}_i = 0$ and equation (29) implies that the elements of $\mathbf{A}_i$ simplify to

$$A_{ij} = \frac{\left(\sigma_{\mu,i}^2 + se_{ij}^2\right)^{-1}}{\sum_{k=1}^{J_i} \left(\sigma_{\mu,i}^2 + se_{ik}^2\right)^{-1}}. \tag{33}$$

Because $\mathbf{B}_i = 0$, equation (28) implies that $g_{ij} = A_{ij}$. The formula in equation (33) is consistent with the weights given by Hedges and Olkin [2, p 199] for a traditional random effects (RE) model with one observation per group. Equation (33) also corresponds to the maximum likelihood estimate of the mean effect size given by Raudenbush [3, p 310 Eq 16.33]. This shows that our more general model, allowing for the possibility of non-zero correlations among sampling errors within groups, is consistent with the traditional RE meta-analysis estimator when considering the special case where the correlations are zero.

To examine the ingredients of the formula that include the sampling error correlations, consider the case with two groups where one of the groups has one observation and the other group has two observations and there are no non-sampling errors within groups, i.e., $\sigma_{\mu,i} = \sigma_{\mu,2} = 0$. In this case, the overall summary estimate is

$$\hat{y} = hy_1 + (1-h)\hat{y}_2 = hy_1 + (1-h)\left[gy_{2,1} + (1-g)y_{2,2}\right], \tag{34}$$

where $\hat{y}_2 = gy_{2,1} + (1-g)y_{2,2}$ is the composite estimate for group 2. With only two groups and three observations, there are only two weights to determine: h and g. We will begin by determining g. The variance of the composite estimate for group 2 is

$$v_2 = g^2 \operatorname{var}\left[y_{2,1}\right] + (1-g)^2 \operatorname{var}\left[y_{2,2}\right] + 2g(1-g)\operatorname{cov}\left[y_{2,1}, y_{2,2}\right]. \tag{35}$$

The first-order condition for an optimum is

$$\frac{\partial v_2}{\partial g} = 2g \operatorname{var}\left[y_{2,1}\right] - 2(1-g)\operatorname{var}\left[y_{2,2}\right] + (2-4g)\operatorname{cov}\left[y_{2,1}, y_{2,2}\right] = 0. \tag{36}$$

substituting ρ and the se's for the covariance and variances gives

$$2gse_{2,1}^2 - 2(1-g)se_{2,2}^2 + (2-4g)\rho se_{2,1}se_{2,2} = 0, \tag{37}$$

which can be solved for g:

$$g = \frac{se_{2,2}^2 - \rho se_{2,1}se_{2,2}}{se_{2,1}^2 + se_{2,2}^2 - 2\rho se_{2,1}se_{2,2}}. \tag{38}$$

S1.5 Stage two

Next we want to find the infeasible optimal weights, h_i, to place on the composite group-level estimates, $\hat{Y}_i$. We will choose the h_i's to minimize the variance of the overall estimate subject to the constraint that the weights sum to one. The variance of the overall estimate is:

$$\operatorname{var}\left[\hat{Y}\right] = \sum\nolimits_{i=1}^{I} h_i^2 v_i, \tag{39}$$

where v_i is the variance of the composite estimate for group i and can be computed using equation (9) after computing (feasible versions of) the g_{ij}'s using equation (31)

from stage one and feasible estimates of the unknown quantities therein. The first-order condition for a minimum is:

$$\frac{\partial \operatorname{var}\left[\hat{Y}\right]}{\partial h_i} = 2h_i v_i - \lambda = 0, \tag{40}$$

where λ is the Lagrange multiplier on the constraint $\sum_{i=1}^{I} h_i = 1$. Solving for h_i gives

$$h_i = \frac{\lambda}{2v_i}. \tag{41}$$

Next, we can use the constraint to write

$$\sum\nolimits_{i=1}^{I} h_i = \sum\nolimits_{i=1}^{I} \frac{\lambda}{2v_i} = 1, \tag{42}$$

then solve for the Lagrange multiplier to get

$$\lambda = \frac{1}{\sum_{i=1}^{I} \frac{1}{2v_i}}, \tag{43}$$

then plug this result back into the expression for the group-level weight, equation (41), to get

$$h_i = \frac{\frac{1}{\sum_{i=1}^{I} \frac{1}{2v_i}}}{2v_i}, \tag{44}$$

which can be simplified by cancelling the 2 and written slightly more compactly as

$$h_i = \frac{v_i^{-1}}{\sum_{i=1}^{I} v_i^{-1}}. \tag{45}$$

This formula for the second-stage group-level weights is directly analogous to that for the within-group observations derived in the first stage—i.e., weights are proportional to the inverse variances of the estimates, where the variances account for contributions from all relevant error components—and so also is consistent with the weights given by Hedges and Olkin [2, p 199] for the traditional RE meta analysis estimator.

S1.6 Estimation of error variances

To construct a feasible version of the two-stage random effects estimator derived above, we require estimates of the unknown error variances. This section derives estimators for the two unknown error variance components: the within-group non-sampling errors ($\sigma^2_{\mu,i}$) and between-group non-sampling errors (σ^2_{η}). At this point we will generalize to a meta-regression framework, so now we assume

$$y_{ij} = x_{ij}\beta + \eta_i + \mu_{ij} + \varepsilon_{ij}. \tag{46}$$

We will begin by ignoring the correlations among sampling errors within groups, then we will generalize our result to allow for non-zero sampling error correlations. If the sampling errors are uncorrelated, then observations in group i can be viewed as drawn from a mixture distribution: with frequency $1/J_i$ the mean is $x_{ij}\beta + \eta_i$ and the variance is $\sigma^2_{\mu,i} + se^2_{ij}$. The variance of a mixture is the weighted average of the variances plus the variance of the means, therefore the expected variance of the observations in group i is

$$\mathbb{E}\left[\operatorname{var}\left[\mathbf{y}_i\right]\right] = \frac{1}{J_i}\sum_{j=1}^{J_i}\left(\sigma^2_{\mu,i} + se^2_{ij}\right) + \operatorname{var}\left[\mathbf{x}_i\beta\right], \tag{47}$$

where $\mathbf{y}_i$ denotes the vector of observations in group i, $\left[y_{i,1}\ \ y_{i,2}\ \ ...\ \ y_{i,J_i}\right]$. We can rearrange equation (47) and use sample estimates of the unknown variance terms to derive a feasible method-of-moments estimator for the non-sampling error variance for group i,

$$\hat{\sigma}^2_{\mu,i} = \mathrm{var}\left[\mathbf{y}_i\right] - \frac{1}{J_i}\sum_{j=1}^{J_i} se^2_{ij} - \mathrm{var}\left[\mathbf{x}_i\hat{\beta}\right]. \tag{48}$$

This estimator is consistent with the estimator derived by Hedges and Olkin [2, p 194] for the across-group non-sampling error variance (i.e., heterogeneity of true effect sizes) in a standard RE model with one observation per group and excluding the $\mathbf{x}_i$ vector. This estimator also appears to be identical to the method-of-moments estimator for the variance of the across-group random effects using OLS regression given by Raudenbush [3, p 311]. Here we are applying the same logic to estimate the variance of the within-group heterogeneity or non-sampling errors.

We derived the estimator in equation (48) under the assumption of uncorrelated sampling errors. With correlated sampling errors, the relevant expression is

$$\hat{\sigma}^2_{\mu,i} = \mathrm{var}\left[\mathbf{y}_i\right] - \frac{1}{J_i}\left[\sum\nolimits_{j=1}^{J_i}\left(se^2_{ij} - \frac{1}{J_i}\sum\nolimits_{k\neq j}^{J_i}\rho_i se_{ij}se_{ik}\right)\right] - \mathrm{var}\left[\mathbf{x}_i\hat{\beta}\right]. \tag{49}$$

This is a generalization of equation (48), here accounting for the covariances among observation-level sampling errors within the group.

It is possible for the estimator in equation (49) to return a negative $\hat{\sigma}^2_{\mu,i}$ when the average of the reported standard errors is greater than the total variance of the individual observations from group i, $\mathrm{var}\left[\mathbf{y}_i\right]$. This can occur due to sampling variability alone especially when J is small. Alternatively, this might suggest a positive correlation between the within-study non-sampling errors; however, we have not included such a correlation term in our estimator. In practice we handle this by setting $\hat{\sigma}^2_{\mu,i} = 0$ in such cases.

Finally, we derive a feasible method-of-moments estimator of σ^2_η based on equation (9):

$$\hat{\sigma}^2_\eta = \frac{1}{I-1}\sum_{i=1}^{I}\left(\hat{y}_i - \tfrac{1}{I}\sum_{i=1}^{I}\hat{y}_i\right)^2 - \frac{1}{I-1}\sum_{i=1}^{I}\sum_{j=1}^{J_i}\left[\hat{g}^2_{ij}\left(\hat{\sigma}^2_{\mu,i} + se^2_{ij}\right) + \rho_i\sum\nolimits_{k\neq j}^{J_i}\hat{g}_{ij}\hat{g}_{ik}se_{ij}se_{ik}\right], \tag{50}$$

where the first term on the right-hand side of equation (50) is the sample analog of v_i in equation (9) and the $\hat{g}_{ij}$'s in the second term are computed by plugging in the estimated values for all unknown quantities in equation (32).

Note that we cannot estimate both the group-level non-sampling error variance $\sigma^2_{\mu,i}$ and the within-group sampling error correlation ρ_i simultaneously for all groups because we have only one equation, expression (50), to identify the two unknowns for each group. To address this limitation, we specify a common value for ρ_i for all groups ex ante and then we estimate the $\sigma^2_{\mu,i}$'s conditional on the maintained assumption about the ρ_i's.

S1.7 Iterative estimation approach

When conducting a meta-regression including one or more explanatory variables, efficient estimation of the coefficient vector $\hat{\beta}$ requires estimates of the observation weights, which in turn requires estimates of the error component variances. However, estimating the error component variances requires an estimate of β, as indicated in the note at the bottom of Table 1 in the main text. This chicken-and-egg problem can be

solved using an iterative estimation approach: Begin by setting all elements of $\hat{\beta}$ to 0 and calculate estimates of the error component variances and observation weights as described in this Appendix and summarized in Table 1 in the main text. Use the resulting weights to perform a weighted least squares regression to produce an updated estimate of β. Use the updated $\hat{\beta}$ to recompute the observation weights, again following the sequence of computations in Table 1 in the main text but now using the adjustment indicated in the note at the bottom of the table. Repeat this process until the estimates change by an amount smaller than a pre-defined tolerance. The stopping criterion used in our illustrative application was when the largest change among the coefficient estimates became smaller than 0.001%, which is safely below the sampling variability of these estimators.

References

1. Hedges LV, Tipton E, Johnson MC. 2010. Robust variance estimation in meta-regression with dependent effect size estimates. *Research Synthesis Methods* 1(1):39–65.

2. Hedges LV, Olkin I. 1985. *Statistical Methods for Meta-Analysis.* Academic Press.

3. Raudenbush SW. 2009. Analyzing effect sizes: random-effects models In *Handbook of Research Synthesis and Meta-Analysis*, edited by Cooper H, hedges LV, Valentine JC. Russel Sage Foundation.
