# Supplementary material for: A two-stage random-effects estimator for meta-analyses of the value per statistical life
Source: PLoS One. 2025 Jun 13;20(6):e0324630. doi: 10.1371/journal.pone.0324630 (PMC12165433; doi:10.1371/journal.pone.0324630)
Supplement: S2 Supplemental tables — Monte Carlo simulation and meta-regression results. (PDF) [file pone.0324630.s003.pdf]

Supporting Information

S2 Supplemental tables The tables in this supporting section show Monte Carlo simulation results for all 16 cases distinguished by the number of groups (I), the number of observations per group (J), the between-group non-sampling error variation (σ_η), and the within-group non-sampling error variation (σ_μ) for all four combinations of ρ and $\hat{\rho}$.

The results in Table S2.1 are based on cases with no correlation among non-sampling errors within groups, $\rho = 0$, and where the analyst has correctly set $\hat{\rho} = 0$. Here the 2SRE-equal estimator is more efficient than the other estimators in 13 out of 16 cases.

The results in Table S2.2 are based on cases with a positive correlation among non-sampling errors within groups, $\rho = 0.5$, and where the analyst has incorrectly set $\hat{\rho} = 0$. Here the 2SRE-equal estimator is more efficient than all other estimators in 12 of 16 cases, the 2SRE-free estimator performs best in one case, and the metafor estimator performs best in the three remaining cases.

The results in Table S2.3 are based on cases with a positive correlation among non-sampling errors within groups, $\rho = 0.5$, and where the analyst has correctly set $\hat{\rho} = 0.5$. Here again the 2SRE-equal estimator is more efficient than all other estimators in all 16 cases.

The results in Table S2.4 are based on cases with no correlation among non-sampling errors within groups, $\rho = 0$, and where the analyst has incorrectly set $\hat{\rho} = 0.5$. Here the 2SRE-equal estimator is as or more efficient than the other estimators in 12 out of 16 cases. Under this $(\rho, \hat{\rho})$ configuration, the robumeta-HIER estimator is most efficient in the remaining 4 cases.

The final columns in Tables S2.1–S2.4 show that the average robust standard errors, $\hat{se}$ [1], which were computed for the 2SRE-equal estimator, closely match the standard deviations of their corresponding simulated estimates, as they should. The R^2 values between the two quantities is greater than 0.99 in all four tables, and we see no apparent bias of the robust standard errors even in Tables S2.2 and S2.4 where $\hat{\rho} \neq \rho$, so it does not appear that mis-specification of $\hat{\rho}$ will substantially compromise standard errors.

Table S2.1 Monte Carlo simulation experiment results ($\rho = 0$, $\hat{\rho} = 0$)

I	J	σ_η	σ_μ	simple mean	group means	meta-for	robum CORR	robum HIER	MAd	2SRE true	2SRE free	2SRE equal	$\bar{se}$
20	1, 5	1.0	0.5,1.0	0.479	0.480	0.405	0.478	**0.378**	0.423	0.366	0.492	0.383	0.392
20	1, 5	1.0	0.5,3.0	0.518	0.534	0.575	0.532	**0.463**	0.499	0.436	0.623	0.465	0.477
20	1, 5	3.0	0.5,1.0	0.834	0.839	0.976	0.824	0.813	0.809	0.771	1.104	**0.774**	0.792
20	1, 5	3.0	0.5,3.0	0.898	0.907	1.176	0.864	0.881	0.842	0.813	0.856	**0.825**	0.859
20	1,15	1.0	0.5,1.0	0.350	0.398	0.319	0.376	0.299	0.338	0.282	0.369	**0.291**	0.304
20	1,15	1.0	0.5,3.0	0.396	0.483	0.438	0.448	0.367	0.387	0.350	0.389	**0.361**	0.371
20	1,15	3.0	0.5,1.0	0.811	0.740	0.837	0.740	0.799	0.727	0.710	0.721	**0.711**	0.740
20	1,15	3.0	0.5,3.0	0.862	0.831	0.940	0.828	0.854	0.804	0.781	0.814	**0.789**	0.772
60	1, 5	1.0	0.5,1.0	0.274	0.298	0.248	0.270	**0.217**	0.252	0.212	0.236	0.220	0.223
60	1, 5	1.0	0.5,3.0	0.300	0.334	0.375	0.316	0.269	0.288	0.253	0.431	**0.267**	0.268
60	1, 5	3.0	0.5,1.0	0.504	0.498	0.596	0.497	0.493	0.485	0.468	0.478	**0.469**	0.442
60	1, 5	3.0	0.5,3.0	0.501	0.505	0.616	0.493	0.489	0.480	0.464	0.482	**0.467**	0.478
60	1,15	1.0	0.5,1.0	0.208	0.248	0.191	0.229	0.177	0.202	0.171	0.182	**0.174**	0.173
60	1,15	1.0	0.5,3.0	0.219	0.245	0.242	0.249	0.204	0.212	0.193	0.245	**0.197**	0.199
60	1,15	3.0	0.5,1.0	0.463	0.431	0.524	0.432	0.462	0.426	0.412	0.418	**0.413**	0.411
60	1,15	3.0	0.5,3.0	0.465	0.446	0.523	0.455	0.467	0.440	0.432	0.455	**0.433**	0.424

Table S2.2 Monte Carlo simulation experiment results ($\rho = 0.5$, $\hat{\rho} = 0$)

I	J	σ_η	σ_μ	simple mean	group means	meta-for	robum CORR	robum HIER	MAd	2SRE true	2SRE free	2SRE equal	$\hat{se}$
20	1, 5	1.0	0.5,1.0	0.629	0.625	**0.464**	0.569	0.481	0.563	0.422	0.473	0.468	0.483
20	1, 5	1.0	0.5,3.0	0.622	0.639	0.530	0.558	0.503	0.571	0.445	0.535	**0.494**	0.496
20	1, 5	3.0	0.5,1.0	0.977	0.893	0.972	0.852	0.894	0.856	0.779	0.807	**0.798**	0.800
20	1, 5	3.0	0.5,3.0	1.015	0.965	1.304	0.957	0.968	0.929	0.879	0.914	**0.893**	0.918
20	1,15	1.0	0.5,1.0	0.642	0.594	**0.402**	0.578	0.470	0.552	0.360	0.425	0.419	0.415
20	1,15	1.0	0.5,3.0	0.535	0.551	0.458	0.523	0.456	0.519	0.385	0.429	**0.430**	0.440
20	1,15	3.0	0.5,1.0	0.894	0.824	0.836	0.832	0.853	0.824	0.732	0.751	**0.743**	0.742
20	1,15	3.0	0.5,3.0	0.909	0.822	0.910	0.815	0.865	0.819	0.745	0.759	**0.757**	0.788
60	1, 5	1.0	0.5,1.0	0.361	0.360	**0.270**	0.335	0.279	0.332	0.240	0.274	**0.270**	0.270
60	1, 5	1.0	0.5,3.0	0.374	0.394	0.369	0.345	0.313	0.338	0.281	0.321	**0.311**	0.320
60	1, 5	3.0	0.5,1.0	0.521	0.489	0.593	0.475	0.505	0.473	0.444	0.456	**0.449**	0.462
60	1, 5	3.0	0.5,3.0	0.547	0.515	0.616	0.515	0.528	0.503	0.479	0.492	**0.484**	0.491
60	1,15	1.0	0.5,1.0	0.333	0.288	0.224	0.283	0.243	0.284	0.191	0.215	**0.210**	0.220
60	1,15	1.0	0.5,3.0	0.339	0.335	0.261	0.317	0.279	0.311	0.226	**0.254**	0.257	0.254
60	1,15	3.0	0.5,1.0	0.537	0.483	0.533	0.484	0.511	0.482	0.430	0.440	**0.436**	0.430
60	1,15	3.0	0.5,3.0	0.555	0.502	0.540	0.495	0.528	0.495	0.452	0.465	**0.460**	0.457

Table S2.3 Monte Carlo simulation experiment results ($\rho = 0.5$, $\hat{\rho} = 0.5$)

I	J	σ_η	σ_μ	simple mean	group means	meta-for	robum CORR	robum HIER	MAd	2SRE true	2SRE free	2SRE equal	$\hat{se}$
20	1, 5	1.0	0.5,1.0	0.629	0.625	0.500	0.577	0.493	0.560	0.436	0.476	**0.454**	0.448
20	1, 5	1.0	0.5,3.0	0.630	0.615	0.726	0.577	0.542	0.575	0.478	0.678	**0.513**	0.510
20	1, 5	3.0	0.5,1.0	0.933	0.871	1.082	0.847	0.886	0.838	0.798	0.818	**0.798**	0.802
20	1, 5	3.0	0.5,3.0	1.017	0.985	1.219	0.973	0.961	0.964	0.898	0.938	**0.907**	0.896
20	1,15	1.0	0.5,1.0	0.598	0.579	0.398	0.566	0.452	0.564	0.355	0.399	**0.362**	0.367
20	1,15	1.0	0.5,3.0	0.620	0.587	0.613	0.550	0.517	0.540	0.406	0.520	**0.433**	0.433
20	1,15	3.0	0.5,1.0	0.909	0.826	0.986	0.822	0.846	0.819	0.731	0.736	**0.730**	0.743
20	1,15	3.0	0.5,3.0	0.971	0.865	1.209	0.863	0.946	0.855	0.803	0.903	**0.806**	0.835
60	1, 5	1.0	0.5,1.0	0.373	0.365	0.300	0.325	0.282	0.319	0.252	0.281	**0.259**	0.257
60	1, 5	1.0	0.5,3.0	0.378	0.368	0.409	0.347	0.311	0.337	0.275	0.310	**0.289**	0.297
60	1, 5	3.0	0.5,1.0	0.520	0.489	0.614	0.484	0.496	0.481	0.438	0.463	**0.439**	0.450
60	1, 5	3.0	0.5,3.0	0.558	0.530	0.723	0.520	0.541	0.519	0.490	0.527	**0.497**	0.499
60	1,15	1.0	0.5,1.0	0.341	0.309	0.220	0.299	0.254	0.290	0.202	0.216	**0.205**	0.205
60	1,15	1.0	0.5,3.0	0.361	0.366	0.313	0.349	0.296	0.339	0.246	0.271	**0.257**	0.252
60	1,15	3.0	0.5,1.0	0.525	0.496	0.496	0.486	0.499	0.483	0.430	0.439	**0.431**	0.423
60	1,15	3.0	0.5,3.0	0.564	0.507	0.592	0.505	0.540	0.504	0.453	0.473	**0.456**	0.450

Table S2.4 Monte Carlo simulation experiment results ($\rho = 0$, $\hat{\rho} = 0.5$)

I	J	σ_η	σ_μ	simple mean	group means	meta-for	robum CORR	robum HIER	MAd	2SRE true	2SRE free	2SRE equal	$\hat{se}$
20	1, 5	1.0	0.5,1.0	0.468	0.537	0.492	0.486	**0.375**	0.453	0.363	0.513	0.382	0.381
20	1, 5	1.0	0.5,3.0	0.503	0.513	0.726	0.498	0.421	0.477	0.398	0.669	**0.417**	0.430
20	1, 5	3.0	0.5,1.0	0.905	0.836	1.164	0.837	0.851	0.821	0.770	0.859	**0.780**	0.795
20	1, 5	3.0	0.5,3.0	0.882	0.883	1.170	0.881	0.847	0.863	0.803	1.026	**0.821**	0.835
20	1,15	1.0	0.5,1.0	0.333	0.341	0.367	0.350	0.287	0.328	0.274	0.371	**0.281**	0.292
20	1,15	1.0	0.5,3.0	0.381	0.482	0.542	0.434	**0.352**	0.436	0.327	0.565	0.362	0.366
20	1,15	3.0	0.5,1.0	0.820	0.745	0.990	0.758	0.803	0.750	0.720	0.960	**0.727**	0.726
20	1,15	3.0	0.5,3.0	0.849	0.761	1.018	0.769	0.832	0.764	0.733	0.782	**0.749**	0.746
60	1, 5	1.0	0.5,1.0	0.299	0.327	0.315	0.306	0.238	0.292	0.231	0.315	**0.235**	0.233
60	1, 5	1.0	0.5,3.0	0.314	0.341	0.479	0.337	**0.276**	0.323	0.257	0.439	0.279	0.284
60	1, 5	3.0	0.5,1.0	0.482	0.469	0.576	0.467	0.463	0.461	0.435	0.663	**0.441**	0.430
60	1, 5	3.0	0.5,3.0	0.471	0.472	0.735	0.470	0.462	0.463	0.449	0.581	**0.456**	0.466
60	1,15	1.0	0.5,1.0	0.199	0.222	0.216	0.222	0.175	0.214	0.166	0.236	**0.171**	0.170
60	1,15	1.0	0.5,3.0	0.233	0.259	0.379	0.262	**0.217**	0.250	0.206	0.312	0.223	0.207
60	1,15	3.0	0.5,1.0	0.461	0.428	0.539	0.432	0.457	0.430	0.412	0.428	**0.414**	0.411
60	1,15	3.0	0.5,3.0	0.446	0.429	0.566	0.428	0.442	0.427	0.419	0.432	**0.426**	0.418

Table S2.5 EPA data meta-regression results with no correction for publication bias. Seven specifications (s0-s6) of the two-stage random-effects meta-regression model with σ_μ unconstrained (2SRE-free) and with *IEVSL* for models including income. Numbers in parentheses are robust standard errors.

	s0	s1	s2	s3	s4	s5	s6
constant	7.612	8.477	9.396	8.324	9.268	9.398	9.055
	(0.615)	(0.870)	(0.731)	(1.153)	(0.961)	(0.690)	(0.922)
SP		-1.382	-2.927	-0.983	-2.600	-2.928	0.065
		(1.368)	(1.374)	(1.954)	(1.701)	(1.356)	(1.412)
median		-0.542	-0.976	-0.686	-1.091	-0.975	-1.731
		(1.383)	(1.286)	(1.403)	(1.322)	(1.301)	(1.008)
year			0.433		0.432	0.435	
			(0.077)		(0.079)	(0.040)	
income				-0.154	-0.124		0.570
				(0.526)	(0.465)		(0.300)
SP×year						-0.002	
						(0.147)	
SP×income							-1.847
							(0.549)
se							
se^2							
σ_μ	2.220	2.216	2.149	2.214	2.146	2.149	2.178
σ_η	2.181	2.183	2.189	2.184	2.190	2.189	2.200
IEVSL				-0.086	-0.069		0.318
				(0.294)	(0.260)		(0.168)
R^2	0.564	0.591	0.681	0.592	0.682	0.681	0.632
R^2_{CV}	0.553	0.549	0.645	0.537	0.634	0.640	0.579

Table S2.6 EPA data meta-regression results with the "precision-effect test" (PET) for publication bias. Seven specifications (s0-s6) of the two-stage random-effects meta-regression model with σ_μ unconstrained (2SRE-free) and with *IEVSL* for models including income. Numbers in parentheses are robust standard errors.

	s0	s1	s2	s3	s4	s5	s6
constant	5.452	5.692	6.929	5.386	6.645	6.809	5.988
	(0.746)	(0.964)	(1.165)	(1.019)	(1.196)	(1.113)	(1.017)
SP		-0.121	-1.528	0.619	-0.865	-1.477	0.986
		(1.126)	(1.233)	(1.466)	(1.459)	(1.250)	(1.371)
median		-0.371	-0.714	-0.628	-0.930	-0.751	-1.111
		(1.149)	(1.106)	(1.172)	(1.147)	(1.125)	(1.123)
year			0.325		0.321	0.281	
			(0.100)		(0.105)	(0.067)	
income				-0.282	-0.244		0.047
				(0.370)	(0.359)		(0.337)
SP×year						0.081	
						(0.147)	
SP×income							-0.839
							(0.576)
se	1.297	1.253	1.005	1.267	1.020	1.017	1.140
	(0.356)	(0.349)	(0.394)	(0.355)	(0.397)	(0.389)	(0.370)
se^2							
σ_μ	2.071	2.066	2.022	2.055	2.012	2.028	2.048
σ_η	2.189	2.192	2.193	2.197	2.197	2.194	2.203
IEVSL				-0.157	-0.137		0.026
				(0.207)	(0.201)		(0.189)
R^2	0.709	0.712	0.754	0.716	0.758	0.754	0.722
R^2_{CV}	0.691	0.674	0.713	0.671	0.708	0.708	0.677

Table S2.7 EPA data meta-regression results with the "precision-effect estimate with SE" (PEESE) for publication bias. Seven specifications (s0-s6) of the two-stage random-effects meta-regression model with σ_μ unconstrained (2SRE-free) and with *IEVSL* for models including income. Numbers in parentheses are robust standard errors.

	s0	s1	s2	s3	s4	s5	s6
constant	6.931	7.494	8.476	7.242	8.258	8.356	7.893
	(0.594)	(0.792)	(0.785)	(0.991)	(0.926)	(0.754)	(0.855)
SP		-0.821	-2.265	-0.177	-1.723	-2.213	0.604
		(1.180)	(1.250)	(1.654)	(1.526)	(1.264)	(1.318)
median		-0.298	-0.715	-0.524	-0.897	-0.753	-1.360
		(1.280)	(1.207)	(1.276)	(1.240)	(1.221)	(1.056)
year			0.374		0.371	0.325	
			(0.084)		(0.087)	(0.063)	
income				-0.248	-0.202		0.311
				(0.457)	(0.427)		(0.324)
SP×year						0.091	
						(0.148)	
SP×income							-1.433
							(0.572)
se							
se^2	0.142	0.130	0.105	0.131	0.107	0.107	0.118
	(0.042)	(0.039)	(0.044)	(0.040)	(0.044)	(0.043)	(0.038)
σ_μ	2.102	2.119	2.098	2.109	2.091	2.094	2.115
σ_η	2.177	2.179	2.185	2.180	2.187	2.186	2.193
IEVSL				-0.138	-0.113		0.174
				(0.256)	(0.239)		(0.181)
R^2	0.660	0.667	0.728	0.671	0.731	0.729	0.692
R^2_{CV}	0.632	0.615	0.676	0.605	0.666	0.672	0.635

Estimates of the constant in Table S2.5 are between \$0.3 and \$0.7 million lower than their counterparts in Table 5 in the main text. Estimates of the coefficient on *se* in Table S2.6 are slightly higher than their counterparts in Table 6 in the main text and now are statistically significant in all specifications, which suggests a stronger signal of publication bias. Estimates of the constant in Table S2.7, which include the PEESE publication bias correction, are between \$0.5 and \$1 million lower than their uncorrected counterparts in Table 7 in the main text. Estimation results shown in Tables S2.5–S2.7 from the 2SRE-free model, with unconstrained group-level non-sampling error variances, $\sigma^2_{\mu,i}$, generally have lower R^2_{CV} values than their constrained counterparts in Tables 5–7 in the main text. This is consistent with our simulation results, which indicate that the 2SRE-equal estimator performs better than the 2SRE-free variant in data environments similar to the preliminary U.S. EPA meta-dataset used in this demonstration application.

References

1. Hedges LV, Tipton E, Johnson MC. 2010. Robust variance estimation in meta-regression with dependent effect size estimates. *Research Synthesis Methods* 1(1):39–65.
