# Supplementary material for: A two-stage random-effects estimator for meta-analyses of the value per statistical life
Source: PLoS One. 2025 Jun 13;20(6):e0324630. doi: 10.1371/journal.pone.0324630 (PMC12165433; doi:10.1371/journal.pone.0324630)
Supplement: S3 Supplemental application — Application to a global VSL meta-dataset. (PDF) [file pone.0324630.s004.pdf]

Supporting Information

S3 Application to a global VSL meta-dataset To our knowledge, the only meta-dataset of global VSL estimates that has been shared publicy by the authors is the dataset compiled by Lindhjem et al. [1], who used meta-regression to analyze 856 VSL estimates from stated preference studies conducted in 38 countries. In their meta-regression, Lindhjem et al. weighted each observation by the inverse of the number of observations contributed by its parent study. Therefore, each study was given equal weight in the meta-regression regardless of the differences in standard errors among the primary estimates or the heterogeneity of the primary estimates within and between studies. To provide a second demonstration of our meta-analysis estimation approach, we applied our 2SRE estimator to the Lindhjem meta-dataset, which we retrieved from the OECD website using the Internet Archive Wayback Machine (`https://web.archive.org`).

We began by attempting to reproduce the basic summary statistics presented in Table 1 of Lindhjem et al., but we were not able to exactly replicate their results. A comparison between Lindhjem et al.'s reported results and our calculations using their data is shown in Table S3.1.

Table S3.1 Comparison between summary statistics reported by Lindhjem et al. (2011) and our attempted replications. All VSL estimates are reported in 2005$US.

	Full sample		Trimmed sample	
	original	replicated	original	replicated
Mean VSL	$6,064,679	$6,026,973	$4,959,587	$4,204,634
Weighted mean VSL	$7,415,484	$7,322,068	$6,314,696	$4,450,844
Median	$2,377,592	$2,381,500	$2,377,592	$2,381,500
Minimum	$4,450	$4,450	$7,292	$50,070
Maximum	$197,000,000	$197,000,000	$86,300,000	$35,720,000
No. of estimates	856	856	814	812

Based on a close examination of Appendix Table A.1 in Lindhjem et al. [1], we were able to identify observations from all included studies that add up to 856, which matches the size of the full sample reported by the authors. However, there are three discrepancies between the archived dataset we downloaded from the OECD website and the results reported by Lindhjem et al.: 1) the study by Andersson was listed in Table A.1 as contributing 8 estimates but only 4 appear in the dataset, 2) the study by Jones-Lee et al. was listed as contributing 18 estimates but 20 appear in the dataset, and 3) the study by Maier et al. was listed as contributing 6 estimates but 8 appear in the dataset. These discrepancies add up to 856 estimates in total, but apparently 6 estimates are different. We note these discrepancies for the sake of completeness and transparency, but they did not impede our use of the Lindhjem data for demonstration purposes.

Results of applying the 2SRE meta-analysis estimator to the Lindhjem data are shown in Table S3.2. After filtering the data to exclude observations without standard errors, 505 estimates from 40 studies remained. In this dataset, the 2SRE-free (unconstrained) and 2SRE-equal (constrained) estimators produce virtually identical mean estimates, both of which are substantially smaller than the simple mean and group mean estimates, which ignore sampling error variances and between- and within-study heterogeneity. The trim-and-fill and especially the PET-PEESE estimates that are designed to correct for publication bias are substantially smaller than the 2SRE estimates. This finding, plus the results of Masterman and Viscusi [2], who compiled a

separate meta-dataset of international VSL estimates from stated preference studies and found that publication biases "account for approximately 90% of the mean value of published VSL estimates in this subset of the literature," suggest that further examination of potential publication bias in this dataset is warranted.

Table S3.2 Meta-analysis estimates of the average global VSL [2005$US] based on the meta-dataset compiled by Lindhjem et al. [1]. Standard errors are based on 1,000 bootstrap re-samples.

Estimator	estimate	s.e.
simple mean	5.22	1.20
group means	6.44	2.51
2SRE–free	3.15	0.67
–equal	3.15	0.66
–free T&F	1.79	0.62
–equal T&F	1.77	0.57
–P-P	0.03	0.17

References

1. Lindhjem H, Navrud S, Braathen NA, Biausque V. 2011. Valuing mortality risk reductions from environmental, transport, and health policies: a global meta-analysis of stated preference studies. *Risk Analysis* 31(9):1381–1407.

2. Masterman CJ, Viscusi WK. 2020. Publication selection biases in stated preference estimates of the value of a statistical life. *Journal of Benefit Cost Analysis* 11(3):357–379.
