# Supplementary material for: A two-stage random-effects estimator for meta-analyses of the value per statistical life
Source: PLoS One. 2025 Jun 13;20(6):e0324630. doi: 10.1371/journal.pone.0324630 (PMC12165433; doi:10.1371/journal.pone.0324630)
Supplement: S4 Data and code — Link to a Github repository containing data and code sufficient to reproduce our results. (PDF) [file pone.0324630.s005.pdf]

Supporting Information

S4 Data and code. A Github repository containing data and a set of `R` scripts sufficient to replicate all results reported in this paper can be found at: `https://github.com/scnewbold/2SRE`.
