# Supplementary material for: A two-stage random-effects estimator for meta-analyses of the value per statistical life
Source: PLoS One. 2025 Jun 13;20(6):e0324630. doi: 10.1371/journal.pone.0324630 (PMC12165433; doi:10.1371/journal.pone.0324630)
Supplement: Attachment — Submitted filename: plos-responsetoreviewers-rev1.pdf [file pone.0324630.s006.pdf]

Response to reviewers

We are grateful to the editor for the opportunity to revise and resubmit our manuscript, and we thank the reviewers for their thoughtful and constructive comments. In this document, we reproduce the reviewers' comments in black text and we provide our response and indicate the changes we made to the manuscript below each comment in blue text.

Reviewer #1: The manuscript titled "A two-stage random-effects estimator for meta-analyses of the value per statistical life" makes a commendable methodological contribution to the field of meta-analysis. The authors address significant challenges in synthesizing results from studies with unbalanced datasets while accounting for different sources of errors. The two-stage random-effects estimator proposed here is robust, rigorously tested using Monte Carlo simulations, and compares favorably against existing methods. The real-world demonstration using a VSL dataset adds practical weight to the study. However, a few refinements can further improve the clarity, accessibility, and impact of the manuscript.

The introduction section, while providing sufficient context, does not explicitly articulate the specific research gap that this study aims to address. While it is clear that the work focuses on improving precision in meta-analysis with unbalanced data, the novelty and objectives of the paper could be stated more clearly upfront. Explicitly stating the shortcomings of existing methods and how this approach resolves those issues will better highlight the importance of the work. Articulating these points will help readers appreciate the methodological advancements and the unique contributions of the proposed estimator.

To make our unique contributions more clear, we added the following passage as the final paragraph before Section 1.1 (starting on line 65):

"We make two main contributions in this study. First, we develop a new multilevel meta-analysis estimator and test its performance against other comparable estimators in a Monte Carlo simulation experiment. Second, we demonstrate the use of our new estimator by applying it to a publicly available meta-dataset of estimates of the value per statistical life (VSL). Our estimation approach can deliver higher precision while accommodating a more general error structure than previous VSL meta-analyses."

To more clearly distinguish our estimation approach from previous multilevel meta-analysis studies, we expanded the opening paragraph of our Methods section to read as follows (starting on line 186):

"Our two-stage random-effects (2SRE) estimation approach is designed to maximize precision using meta-data that take the form of balanced or unbalanced panels, which means it can accommodate primary studies that contribute any number of observations. It also accounts for sampling and non-sampling sources of error both within and across studies. Our approach is related to other multilevel meta-analysis methods, including the hierarchical dependence model described by Hedges et al. (2010) and Tipton (2015) and the three-level meta-analysis approach

described by Konstantopoulos (2011). However, in contrast to the studies by Hedges et al. and Tipton, which focus on robust variance estimation for multilevel models given any weighting scheme, our approach uses the metadata to estimate efficient weights. And in contrast to the study by Konstantopolous, which uses a maximum likelihood approach to estimate a single within-study residual error variance, our approach uses the method of moments to estimate non-sampling error variances for each study without assuming a parametric form for the error distributions. Our methodological contributions include: 1) tailoring a multilevel random-effects estimator to features that we expect to characterize many VSL meta-datasets, 2) conducting a series of Monte Carlo simulation experiments to examine the performance of the estimator in comparison to several other commonly used meta-analysis estimators in our data environment, and 3) applying the estimator to a preliminary meta-dataset of VSL estimates from revealed and stated preference studies conducted in the United States between 1999 and 2019."

The dataset used for demonstration, although well-documented and appropriate for testing the proposed method, is somewhat dated, spanning studies from 1999 to 2013 and limited to the U.S. context. While the methodological focus of the paper is clear, the reliance on older data may raise concerns regarding the broader relevance and applicability of the results. It would strengthen the paper if the authors explicitly acknowledged this limitation and discussed how applying the method to a more recent or globally diverse dataset might influence the findings. A short note suggesting this as future work would also enhance the study's scope.

We were able to find one newer primary study that estimated the VSL using U.S. data. Lee and Taylor (2019) reported 25 estimates of the VSL, so this increased the number of included studies in our meta-dataset from 18 to 19 and the number of primary estimates from 88 to 113. All results and associated discussion points throughout the manuscript have been updated accordingly. Also, because Lee and Taylor used a quasi-experimental approach that is related to but distinct from traditional hedonic wage methods, most references to "hedonic wage (HW)" studies have been replaced with "revealed preference (RP)" studies.

To acknowledge the limitation that our dataset includes only U.S. estimates, we added the following passage as the penultimate paragraph in the Discussion section (starting on line 636):

"A practical limitation of our application is that our meta-dataset includes only U.S. estimates of the VSL. For policy analyses in other countries, at least two options are available: 1) conduct a separate meta-analysis using domestic VSL estimates, or 2) adjust a U.S. VSL estimate to account for income differences between countries, as recommended by Viscusi and Masterman (2017). For instance, Lindhjem et al. (2011) used meta-regression to analyze 856 VSL estimates from stated preference studies conducted in 38 countries. While a comprehensive reanalysis of Lindhjem's data is beyond the scope of this paper, we applied our 2SRE estimator to their meta-dataset in a side analysis. In section S3 of the Supporting Information, we compare and contrast our results to the raw mean and the study-weighted mean VSL reported by Lindhjem et al. This demonstrates that our estimation approach can be effectively applied to other VSL meta-datasets that include both point estimates and standard errors without practical barriers."

We also added the following section to the Supporting Information (starting near the bottom of page 40):

S3 Application to a global VSL meta-dataset To our knowledge, the only meta-dataset of global VSL estimates that has been shared publicy by the authors is the dataset compiled by Lindhjem et al. (2011), who used meta-regression to analyze 856 VSL estimates from stated preference studies conducted in 38 countries. In their meta-regression, Lindhjem et al. weighted each observation by the inverse of the number of observations contributed by its parent study. Therefore, each study was given equal weight in the meta-regression regardless of the differences in standard errors among the primary estimates or the heterogeneity of the primary estimates within and between studies. To provide a second demonstration of our meta-analysis estimation approach, we applied our 2SRE estimator to the Lindhjem meta-dataset, which we retrieved from the OECD website using the Internet Archive Wayback Machine.[1]

We began by attempting to reproduce the basic summary statistics presented in Table 1 of Lindhjem et al., but we were not able to exactly replicate their results. A comparison between the results reported by Lindhjem et al. and our calculations using their data is shown in Table S3.1.

Table S3.1 Comparison between summary statistics reported by Lindhjem et al. (2011) and our attempted replications. All VSL estimates are reported in 2005$US.

	Full sample		Trimmed sample	
	original	replicated	original	replicated
Mean VSL	$6,064,679	$6,026,973	$4,959,587	$4,204,634
Weighted mean VSL	$7,415,484	$7,322,068	$6,314,696	$4,450,844
Median	$2,377,592	$2,381,500	$2,377,592	$2,381,500
Minimum	$4,450	$4,450	$7,292	$50,070
Maximum	$197,000,000	$197,000,000	$86,300,000	$35,720,000
No. of estimates	856	856	814	812

Based on a close examination of Appendix Table A.1 in Lindhjem et al. (2011), we were able to identify observations from all included studies that add up to 856, which matches the size of the full sample reported by the authors. However, there are three discrepancies between the archived dataset we downloaded from the OECD website and the results reported by Lindhjem et al.: 1) the study by Andersson was listed in Table A.1 as contributing 8 estimates but only 4 appear in the dataset, 2) the study by Jones-Lee et al. was listed as contributing 18 estimates but 20 appear in the dataset, and 3) the study by Maier et al. was listed as contributing 6 estimates but 8 appear in the dataset. These discrepancies add up to 856 estimates in total, but apparently 6 estimates are different. We note these discrepancies for the sake of completeness and transparency, but they did not impede our use of the Lindhjem data for demonstration purposes.

[1] `https://web.archive.org`

Results of applying the 2SRE meta-analysis estimator to the Lindhjem data are shown in Table S3.2. After filtering the data to exclude observations without standard errors, 505 estimates from 40 studies remained. In this dataset, the 2SRE-free (unconstrained) and 2SRE-equal (constrained) estimators produce virtually identical mean estimates, both of which are substantially smaller than the simple mean and group mean estimates, which ignore sampling error variances and between- and within-study heterogeneity. The trim-and-fill and especially the PET-PEESE estimates that are designed to correct for publication bias are substantially smaller than the 2SRE estimates. This finding, plus the results of Masterman and Viscusi (2020), who compiled a separate meta-dataset of international VSL estimates from stated preference studies and found that publication biases "account for approximately 90% of the mean value of published VSL estimates in this subset of the literature," suggest that further examination of potential publication bias in this dataset is warranted.

Table S3.2 Meta-analysis estimates of the average global VSL [2005$US] based on the meta-dataset compiled by Lindhjem et al. (2011). Standard errors are based on 1,000 bootstrap re-samples.

Estimator	estimate	s.e.
simple mean	5.22	1.20
group means	6.44	2.51
2SRE–free	3.15	0.67
–equal	3.15	0.66
–free T&F	1.79	0.62
–equal T&F	1.77	0.57
–P-P	0.03	0.17

The paper lacks a formal conclusion section, which leaves the manuscript feeling incomplete. While the results and discussion are thorough, a concise summary of the key contributions, findings, and implications would provide a stronger closure to the work. Including a brief conclusion that reiterates the main takeaways, such as the methodological advancements, the precision of the new estimator, and its comparative performance, would help solidify the impact of the study. Additionally, outlining areas for future research or potential applications of the estimator would add value for readers seeking next steps.

In our previous draft, we used the IMRaD (Introduction, Methods, Results, and Discussion) format that its common for many scientific journals. Nevertheless, we agree with the reviewer that a dedicated section with a summary and concluding thoughts will help to complete the paper. Therefore, we removed the final paragraph of our Discussion section and added a dedicated Conclusions section (starting at line 656):

"In this study, we developed a two-stage random-effects meta-analysis estimator designed to handle unbalanced panels with one or multiple observations from each independent group of primary estimates. The method separates sampling from non-sampling sources of error within

and between groups, leveraging the data to calculate efficient weights for clustered observations. We examined the performance of the estimator through a series of Monte Carlo experiments on constructed datasets. The simulation results demonstrate that the estimator performs well compared to several commonly used meta-analysis methods, including other multilevel estimators, particularly in scenarios with modest dataset sizes (20 to 60 studies, each contributing 1 to 15 observations) and significant heterogeneity within and between studies. At the tested sample sizes, the method is most effective when within-group non-sampling error variances are assumed to be homogeneous across groups. To illustrate the approach, we applied it to a meta-dataset comprising 113 value per statistical life (VSL) estimates derived from 10 revealed preference and 9 stated preference studies conducted in the United States between 1999 and 2019. Future meta-analyses in many domains—including but not limited to analyses of the VSL—may have similar data characteristics, so the usefulness of the 2SRE estimation approach or others like it should be correspondingly broad. Our findings suggest that wider adoption and further refinement of meta-analysis techniques, including multilevel estimators like the one developed here, can enhance the accuracy and precision of policy analyses. These advances are critical for synthesizing multiple estimates of policy-relevant metrics, including the VSL and many others."

While the focus on methodological rigor is appreciated, the paper does not discuss practical applications or use cases where the proposed estimator could influence real-world decision-making. The study would benefit from a brief discussion of how more precise VSL estimates could improve cost-benefit analyses in areas such as public health, safety regulations, or environmental policy. Providing even a hypothetical example would help readers, especially policymakers or applied researchers, see the broader implications and practical value of the estimator. This would extend the paper's relevance beyond academia and highlight its utility in shaping policy outcomes.

To address this comment, we added the following brief discussion as the penultimate paragraph of Section 1 (starting on line 52):

"Precise estimates of the VSL play a critical role in informing benefit-cost analyses for policies aimed at improving public health, safety, and environmental quality. For example, in public health, VSL estimates can be used to guide investments in vaccination programs by quantifying the monetary benefits of saved lives, ensuring that resources are allocated efficiently (Watts et al. 2021). In safety regulations, more precise VSL estimates could help set standards for automobile safety features, such as airbags or collision avoidance settings for driverless cars (Takaguchi et al. 2022), that better balance their costs with the benefits of reducing traffic fatalities (Milligan et al. 2014). Similarly, in environmental policy, VSL estimates often influence decisions on pollution control measures, such as setting emissions limits for factories (Cropper et al. 2011). These applications demonstrate how robust meta-analytic methods can bolster evidence-based policymaking and maximize the net benefits of public health and environmental regulations by providing more precise estimates of the key factors that drive policy evaluations."

The technical nature of the paper, particularly in the methods and discussion sections, makes it inaccessible to non-specialist readers or students new to the subject. While the intended

audience may be researchers and statisticians, simplifying parts of the discussion to include a plain-language summary of the findings would significantly improve accessibility. Briefly explaining the estimator's benefits in non-technical terms and summarizing why it matters for meta-analysis would broaden the paper's appeal to students, applied researchers, and practitioners.

We hope that the new paragraph we added to the Introduction section (starting on line 52) will be partially responsive to this comment. For the benefit of those readers who may choose to skim or skip the technical details in our Methods section, we also added the following short discussion just before Section 2 (starting on line 170):

"To set the stage for the technical details of our proposed estimation approach in the following section, we conclude this section by restating the aims and potential benefits of our study. We have developed a new method for synthesizing quantitative estimates from the scientific literature, designed to handle scenarios where studies contribute varying numbers of estimates for the same policy-relevant parameter—a common occurrence in real-world research. By carefully distinguishing between different sources of uncertainty in the data, this method helps to ensure that the final weighted mean estimate is as accurate and precise as possible. Why is this important? For those who rely on meta-analyses to inform or evaluate public policies—such as researchers, policymakers, or students—our method offers a more reliable way to draw meaningful insights from diverse studies. In areas like public health, safety regulations, and environmental policy, decision-makers often use these summaries to assess costs and benefits. More precise and reliable meta-analysis estimators enable smarter, evidence-based decisions, whether determining air quality standards, improving road safety measures, or addressing challenges in other critical domains."

The publication bias results, although tested with Trim-and-Fill and PET-PEESE methods, appear underemphasized in the paper. Since publication bias is a key concern in meta-analyses, summarizing these findings more clearly and discussing their implications for VSL estimates would provide additional insight. A short paragraph within the results or discussion section addressing this point would suffice.

The modest emphasis on our publication bias results was deliberate, as our primary contribution lies in enhancing the precision of meta-analysis estimators rather than addressing publication bias or other forms of selection bias. To help manage readers' expectations, we added the following parenthetical disclaimer in Section 2.1 (starting on line 259):

"(While publication bias is an important issue, it is not the primary focus of our methodological contribution in this study; as such, our discussion of it will remain limited.)"

We also added the following footnote at the end of Section 2.3 (at the bottom of page 11) to further signal the importance of this issue:

"Newer methods for detecting and addressing publication bias have been proposed (e.g., An-

drews and Kasy 2019, Lin and Chu 2018). Integrating our meta-analysis estimator with these approaches is a more complex task that we do not attempt here, so we flag this as an important direction for future research."

Finally, while the figures and tables are helpful, some of them—such as the Monte Carlo simulation results—are dense and may be challenging for readers to interpret. Enhancing the figures with clearer labels, brief annotations, or takeaway messages would make the results more intuitive and engaging. Minor language refinements for flow and readability would also improve the overall presentation of the paper.

We appreciate this suggestion. On reflection, we believe that our terse captions for Figure 1 and Table 4 were the most egregious. To make it more comprehensible, we changed the caption for Figure 1 from this,

"Box center lines are average and top and bottom edges are max and min $(\hat{se} - se_{min}) / se_{min}$—so lower heights indicate better performance—over the 16 experimental combinations examined in the Monte Carlo simulation experiments."

to this,

"The center, bottom, and top horizontal lines of each box are the average, minimum, and maximum relative precision measures for each estimator across the 16 experimental design settings tested. The relative precision was computed as the difference between the standard error of the estimator and the minimum theoretical standard error divided by the minimum theoretical standard error, i.e., $(\hat{se} - se_{min}) / se_{min}$. Therefore, lower box heights indicate better performance."

And to make it more self-contained, we expanded the caption for Table 4 to read as follows:

"Meta-analysis estimates of the average VSL among US adults [2020$US] based on the preliminary meta-dataset compiled by the USEPA (2016). Four estimates with the lowest RMSE's are highlighted in bold font. "mm" rows indicate datasets including both mean and median primary estimates. "m" rows indicate datasets including only mean primary estimates. The "RP" column indicates datasets including only revealed preference estimates, the "SP" column indicates datasets including only stated preference estimates, the "pooled" column includes both RP and SP estimates, and the "balanced" column includes both types of estimates with equal weight placed on each."

In conclusion, the authors have made a significant methodological advancement in meta-analysis that is both rigorous and innovative. The study addresses long-standing limitations in unbalanced datasets and error decomposition, and the proposed two-stage random-effects estimator is robustly tested. With clearer articulation of the research gap, an updated conclusion, practical use case discussions, and improved accessibility for broader audiences, this paper has the potential to make an even greater impact.

Reviewer #2: Add diagrams to illustrate the method. This may help readers better understand the context and estimation steps.

We added a new Figure 1 just before Section 2.2 (starting after line 313). The figure shows a flow diagram of estimation steps for the meta-regression estimator, which requires iteration to convergence of the moderator coefficients, β.

Figure 1: Steps required to compute the two-stage random-effects meta-regression estimator, with moderator variables x, using iterated least squares. Detailed equations are presented in Table 2. For meta-analysis, with no moderator variables, step (6) involves computing the overall weighted mean, $\hat{Y}$, rather than estimating the moderator coefficients, β, and no iteration is required.

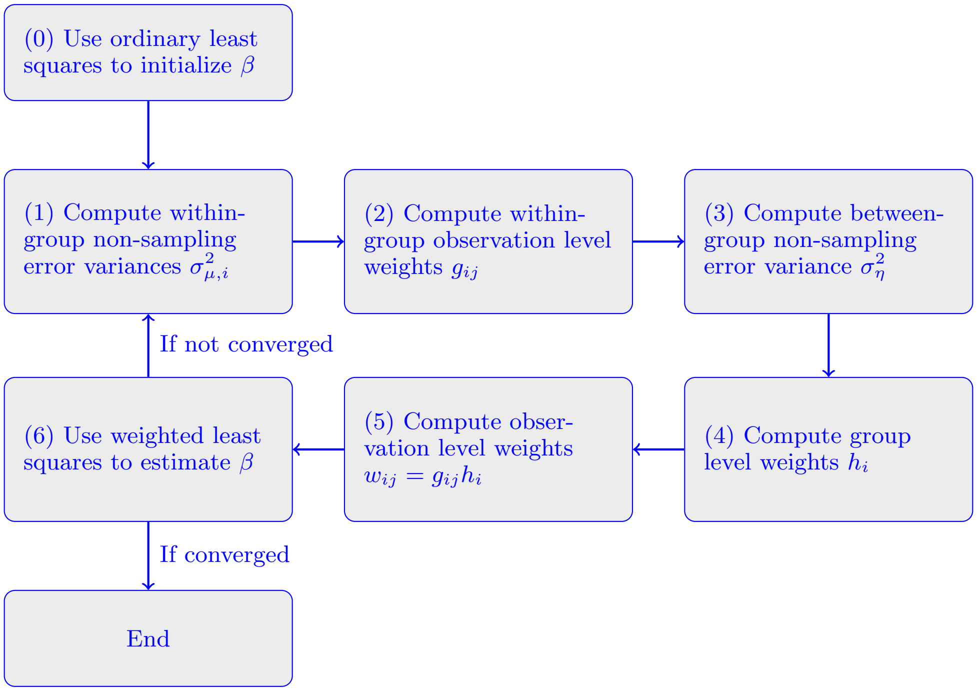

Complement the article with an analysis of a more recent and global data set, including studies after 2020, especially estimates for the COVID-19 period.

We were able to add 25 additional estimates of the VSL to our demonstration dataset from the study by Lee and Taylor (2019). For details, see our response to the related comment by Reviewer #1 starting on page 2 above.

We could find no new estimates of the VSL published since 2019 that satisfy our selection criteria, so unfortunately we are not able to examine any changes in the average VSL since the COVID-19 period.

The article focuses basically on the United States and does not discuss the applicability of the method in other countries.

We were able to download and re-analyze the meta-dataset of global VSL estimates assembled by Lindhjem et al. (2011). For details, see our response to the related comment by Reviewer #1 starting on page 2 above.

The specific benefits should be emphasized that the new estimation method could bring when designing regulations such as health, environmental or road safety.

To address this comment, we added the following brief discussion as the penultimate paragraph in Section 1 (starting on line 52):

"Precise estimates of the VSL play a critical role in informing benefit-cost analyses for policies aimed at improving public health, safety, and environmental quality. For example, in public health, VSL estimates can be used to guide investments in vaccination programs by quantifying the monetary benefits of saved lives, ensuring that resources are allocated efficiently (Watts et al. 2021). In safety regulations, more precise VSL estimates could help set standards for automobile safety features, such as airbags or collision avoidance settings for driverless cars (Takaguchi et al. 2022), that better balance their costs with the benefits of reducing traffic fatalities (Milligan et al. 2014). Similarly, in environmental policy, VSL estimates often influence decisions on pollution control measures, such as setting emissions limits for factories (Cropper et al. 2011). These applications demonstrate how robust meta-analytic methods can bolster evidence-based policymaking and maximize the net benefits of public health and environmental regulations by providing more precise estimates of the key factors that drive policy evaluations."

Expand your explanation of the differences between methods and provide recommendations for when to use each technique.

We understand this comment to pertain mainly to the differences between the 2SRE-free and 2SRE-equal variants of our estimator. We are reluctant to be too prescriptive about this because, while the 2SRE-equal estimator clearly outperformed the 2SRE-free variant in our Monte Carlo experiments, we would not rule out the reverse outcome in larger datasets with more heterogeneity among primary studies. Nevertheless, we added a slightly stronger recommendation about this in the Discussion section (starting on line 583):

"We believe $\rho = 0.5$ is more realistic than $\rho = 0$, so we would recommend $\hat{\rho} = 0.5$ as a default setting and the 2SRE-equal variant as a default model due to its superior performance in the range of data environments examined here."
