# Supplementary material for: A two-stage random-effects estimator for meta-analyses of the value per statistical life
Source: PLoS One. 2025 Jun 13;20(6):e0324630. doi: 10.1371/journal.pone.0324630 (PMC12165433; doi:10.1371/journal.pone.0324630)
Supplement: Attachment — Submitted filename: plos-responsetoreviewers-rev2.pdf [file pone.0324630.s007.pdf]

Response to reviewers

We are grateful to the editor for another opportunity to revise and resubmit our manuscript, and we thank the reviewers for reacting to our first round of revisions. Below, we reproduce the reviewers' comments in black text and we provide our responses to reviewer 3's new comments in blue text.

Reviewers' comments:

Reviewer's Responses to Questions Comments to the Author

1. If the authors have adequately addressed your comments raised in a previous round of review and you feel that this manuscript is now acceptable for publication, you may indicate that here to bypass the "Comments to the Author" section, enter your conflict of interest statement in the "Confidential to Editor" section, and submit your "Accept" recommendation.

Reviewer #1: All comments have been addressed

Reviewer #2: All comments have been addressed

Reviewer #3: All comments have been addressed

2. Is the manuscript technically sound, and do the data support the conclusions?

The manuscript must describe a technically sound piece of scientific research with data that supports the conclusions. Experiments must have been conducted rigorously, with appropriate controls, replication, and sample sizes. The conclusions must be drawn appropriately based on the data presented.

Reviewer #1: Yes

Reviewer #2: Yes

Reviewer #3: Partly

3. Has the statistical analysis been performed appropriately and rigorously?

Reviewer #1: Yes

Reviewer #2: Yes

Reviewer #3: Yes

4. Have the authors made all data underlying the findings in their manuscript fully available?

The PLOS Data policy requires authors to make all data underlying the findings described in their manuscript fully available without restriction, with rare exception (please refer to the Data Availability Statement in the manuscript PDF file). The data should be provided as part of the manuscript or its supporting information, or deposited to a public repository. For example, in addition to summary statistics, the data points behind means, medians and variance measures should be available. If there are restrictions on publicly sharing data—e.g. participant privacy or use of data from a third party—those must be specified.

Reviewer #1: Yes

Reviewer #2: Yes

Reviewer #3: Yes

5. Is the manuscript presented in an intelligible fashion and written in standard English?

PLOS ONE does not copyedit accepted manuscripts, so the language in submitted articles must be clear, correct, and unambiguous. Any typographical or grammatical errors should be corrected at revision, so please note any specific errors here.

Reviewer #1: Yes

Reviewer #2: Yes

Reviewer #3: Yes

6. Review Comments to the Author

Please use the space provided to explain your answers to the questions above. You may also include additional comments for the author, including concerns about dual publication, research ethics, or publication ethics. (Please upload your review as an attachment if it exceeds 20,000 characters)

Reviewer #1: Thank you for your thorough revisions and thoughtful responses to the reviewer comments. The manuscript has significantly improved in clarity, structure, and methodological rigor. The research gap and novelty are now well-articulated, the discussion has been expanded to provide stronger comparisons with past studies, and the addition of a conclusion section has strengthened the overall presentation. The acknowledgment of dataset limitations and the refinement of methodological explanations further enhance the study's credibility. While minor areas such as dataset freshness and additional real-world applications could still be refined, they do not impact the core validity of the study. Given these improvements, I recommend accepting the manuscript with no further major revisions.

Reviewer #2: The authors have taken note of the observations and incorporated them into the article. I recommend the study for publication in this form.

Reviewer #3: The authors aim in their work to develop and present "A two-stage random-effects estimator for meta-analyses of the value per statistical life".

Overall, the work was well done and presented comprehensively.

A brief comment: on line 160, there is a double "and"... it should simply be "and 3)...".

We have double-checked our manuscript to ensure that no instances of "and and" appear near line 160 or elsewhere.

I also suggest that the authors provide a graphical abstract to better understand the manuscript.

We appreciate this prompt from the reviewer. We created the following graphical abstract to accompany our paper. (This is new terriroty for us, so we are open to suggestions for improvements from the reviewers or the editor.)

We developed a **two-stage meta-analysis estimator** for synthesizing effect sizes published in independent studies. The primary estimates from each study are used to compute study-level means, then the study-level means are used to compute an overall mean. Sampling error variances (based on standard errors reported in each study) and non-sampling error variances (estimated from the meta-data) are used to compute efficient weights for combining the estimates.

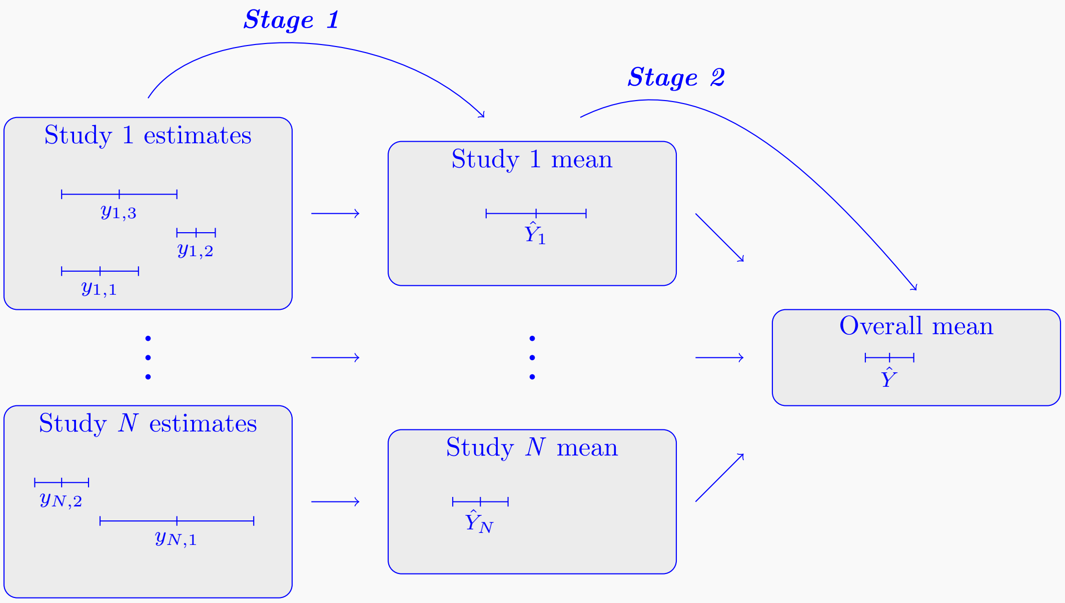

We used simulation tests to compare the performance of our estimator to other commonly used meta-analysis methods, and we demonstrated our approach in an application to a real meta-dataset including 113 estimates of the **value per statistical life** (VSL) drawn from 19 published studies.

However, how confident can the authors be that their new estimator performs similarly when applied to data from sources other than the United States? Is this performance similar to or significantly better than that of known estimators? It would be helpful for the authors to validate their methodology by applying this method to a database from a country similar to the United States and from a much more distant country (a developing country).

To answer my question, the authors have the choice between applying their method to two additional datasets for validation purposes (one relating to a European country and the other to a developed country) or to enrich the discussion in their article. I much prefer the first option.

We are not aware of any publicly available VSL meta-datasets that focus on developing countries. In lieu of a new analysis, we call the reviewer's attention to the analysis we added in our prior revision involving the global meta-dataset of VSL estimates assembled by Lindhjem et al. Their dataset includes VSL estimates from the following 30 countries (plus the EU): Austria, Bangladesh, Belgium, Brazil, Cambodia, Canada, Chile, China, Chinese Taipei

(Taiwan), Czech Rep, Denmark, EU, France, Germany, Hungary, India, Italy, Japan, Korea, Malaysia, Netherlands, New Zealand, Norway, Poland, Spain, Sweden, Switzerland, Thailand, United Kingdom, and the United States. Many of these are developed countries, but a half dozen or more could be considered as still developing or emerging industrialized economies. Economic conditions in the origin countries might affect which primary non-market valuation methods are best suited to estimate the VSL there, but we do not believe they would influence the use of meta-analysis to synthesize the resulting primary estimates. However, if typically fewer primary VSL estimates are available from developing countries, then this would tilt the balance toward simpler meta-analysis methods. That choice and similar operational decisions should be made on a case-by-case basis. Overall, while we cannot provide guarantees that our estimator will perform better than the alternatives in all settings, we have enough confidence to recommend that it be added to meta-analysts' tool kits for consideration in many settings, including applications in developing countries.

We also believe that the best way to assess the performance of statistical estimators is through simulation studies where the true parameters of the data generating process are known. This was the main goal of the simulation experiments described in sections 2.2 and 3.1 of our manuscript. Our findings in section 3.1 should apply to any meta-dataset—including estimates of the VSL or any other effect sizes, from developed or developing countries—that fall within the space of characteristics that we examined in our Monte Carlo experiments.

As a partial response to the reviewer's comment, we added a new citation and modified our brief discussion of the Lindhjem dataset as follows (starting at line 642):

"While to date there are limited or no data estimating the VSL in most low- and middle-income countries (Redfern et al. 2024), our re-analysis of the Lindhjem data demonstrates that our estimation approach can be effectively applied to other meta-datasets of the VSL (or other effect sizes of interest) that include both point estimates and standard errors without practical barriers."

7. PLOS authors have the option to publish the peer review history of their article (what does this mean?). If published, this will include your full peer review and any attached files.

If you choose "no", your identity will remain anonymous but your review may still be made public.

Do you want your identity to be public for this peer review? For information about this choice, including consent withdrawal, please see our Privacy Policy.

Reviewer #1: No

Reviewer #2: No

Reviewer #3: No
